# A Comparative Study of Shade-Matching Reproducibility Using an Intraoral Scanner and a Spectrophotometer

**DOI:** 10.3390/dj12030062

**Published:** 2024-03-04

**Authors:** Franciele Floriani, Carlos A. Jurado, Salahaldeen Abuhammoud, Marcos Vargas, Nicholas G. Fischer, Silvia Rojas-Rueda, Guilherme Carpena Lopes

**Affiliations:** 1 Department of Prosthodontics, The University of Iowa College of Dentistry and Dental Clinics, Iowa City, IA 52242, USA; 2 Division of Operative Dentistry, Department of General Dentistry, The University of Tennessee Health Science Center College of Dentistry, Memphis, TN 38103, USA; 3 Department of Family Dentistry, The University of Iowa College of Dentistry and Dental Clinics, Iowa City, IA 52242, USA; 4 Minnesota Dental Research Center for Biomaterials and Biomechanics, University of Minnesota School of Dentistry, Minneapolis, MN 55108, USA; 5 School of Dentistry, Universidad Javeriana, Bogota 110231, Colombia; 6 College of Dentistry, Federal University of Santa Catarina, Florianopolis 88040-900, SC, Brazil

**Keywords:** shade matching, color, color stability, intraoral scanner, spectrophotometer

## Abstract

Background: We compared the repeatability of the shade determination of resin composite restorations and acrylic teeth in light and darker shades at baseline and after an aging process through two digital tooth color-matching methods: using a Trios 3Shape intraoral scanner and using a Vita Easyshade Compact spectrophotometer. Material and Methods: Forty upper central incisor acrylic teeth in the shade A1 (*n* = 10) and A3 (*n* = 10) were randomly assigned to be restored with Filtek Bulk Fill in the shade A1 (*n* = 10) or A3 (*n* = 10). Subsequently, 20 Class V cavities were prepared in a standardized manner (mesio-distal = 3.0 mm, cervical–occlusal = 2.0 mm, depth = 1.5 mm). Cavities were restored using an universal adhesive system and resin composite in two increments and were light-cured. The shade difference between the resin composite Class V restorations in acrylic teeth of the A1 and A3 shades was evaluated at baseline and after aging. Aging was simulated using ultraviolet light for 120 h. An Easyshade device and an intraoral scanner were used under D65 illumination. Measurements were taken five times, on top of the restoration and on the acrylic teeth, in a randomized manner. Results: Data analysis was on the calculation of the arithmetic mean for the percentage of repeatability conducted by the Trios scanner and the Easyshade device. There was no statistically significant comparison between the shade measurement devices (*p* > 0.05). At baseline, the repeatability for both the Trios intraoral scanner and the Vita Easyshade Compact device for artificial teeth in the shades A1 and A3 was 100%. After aging, the trueness recorded by the intraoral scanner and the Easyshade device for artificial teeth in the shade A1 was 80%. For Class V restoration with shade A1, the intraoral scanner recorded 80% trueness and the Easyshade device recorded 60% trueness at baseline. For shade A3, the intraoral scanner recorded 60% trueness and the Easyshade device recorded 60% trueness. Conclusions: The intraoral scanner and Easyshade device are reliable for baseline shade selection, but their accuracy decreases after aging, particularly for darker shades.

## 1. Introduction

Accurate tooth shade matching in restorations plays a crucial role in patient acceptance and satisfaction, as it directly influences the final appearance of the restoration and its integration with the surrounding teeth [1]. However, tooth shade matching poses significant challenges for dentists and dental technicians [2,3,4]. Tooth shade evaluation employs visual assessment and instrumental techniques [5]. The optical method utilizes conventional shade guides and relies on the unaided eye for shade matching [3,5]. In this context, the shade rings of the manufacturer VITA, including the Vita Classical A1-D4 and the VITA 3D-MASTER (VITA Zahnfabrik, Bad Sackingen, Germany), are well established in the dental market. Despite its popularity, this method suffers from subjectivity and inconsistency [6,7]. Factors such as lighting conditions, the operator’s experience [8,9,10,11], and discrepancies between shade guides and restorative materials [12] complicate the visual method.

Conversely, instrumental methods offer greater precision based on using spectrophotometry and colorimetry [5]. The Vita Easyshade Compact (Vita North America, Yorba Linda, CA, USA) device, a popular handheld and wireless spectrophotometer, has shown superior results compared to other devices [7], with higher precision and reliability in both in vitro and in vivo tests [13]. However, spectrophotometry is often expensive, complex, and operator-dependent [14]. Environmental factors such as light shade and intensity, background, and equipment quality can impact spectrophotometric analysis [15,16,17,18]. Overall, accurately measuring tooth shade in a clinical setting remains challenging [7,19]. In order to address these challenges, an alternative method based on the analysis of standardized digital tooth photographs has gained popularity over the past fifteen years [20]. Despite these improvements, digital images, captured under standardized lighting conditions, may serve as a reliable alternative method for direct shade matching. The ISM method requires a camera, related accessories, and computer skills. However, as these tools are commonly available nowadays, the focus for practitioners should be on effectively learning and applying these tools to achieve the best results [21].

With the advancement of digital technology in dentistry, clinicians have the opportunity to work in a virtual environment, enhancing diagnosis, planning, and treatment [22]. The Trios 3Shape intraoral scanner features a shade selection capability, enabling shade determination across different regions of a tooth’s clinical crown from digital impressions [22]. Previous studies have suggested the Trios 3Shape intraoral scanner as a viable alternative to the Vita Easyshade Compact device for shade determination [21]. However, the efficacy of intraoral digital scanners with integrated shade detection functions as substitutes for colorimeters or spectrophotometers is not fully established [20]. Previous clinical studies revealed discrepancies between Trios 3Shape intraoral scanner shade matching and spectrophotometric analysis [20,21,22]. In the literature, no one has evaluated shade matching in resin composite restorations or in acrylic teeth using the Trios 3Shape intraoral scanner shade measurement method.

Therefore, the aim of this study is to compare the repeatability of the shade selection of resin composite restorations and acrylic teeth in lighter and darker shades at baseline and after undergoing an aging process using two digital tooth color-matching devices: the Trios 3Shape intraoral scanner and the Vita Easyshade Compact device. The hypothesis is that (1) shade matching in acrylic teeth and resin composites will not be significantly influenced by the method of analysis; (2) shade matching in acrylic teeth and resin composites will be significantly influenced by the method of shade analysis.

## 2. Materials and Methods

The sample size was determined based on a prior study and was conducted using the G*Power software (Statistical Package for the Social Sciences, SPSS version 25, Chicago, IL, USA), aiming for a power of 80% and a significance level of 5%. The sample size calculation indicated a need for 40 samples of acrylic teeth in the shades A1 (*n* = 10) and A3 (*n* = 10) restored with Filtek Bulk Fill in the shade A1 (*n* = 10) or A3 (*n* = 10) [12]. 

### 2.1. Specimen Preparation

**Figure 1:**
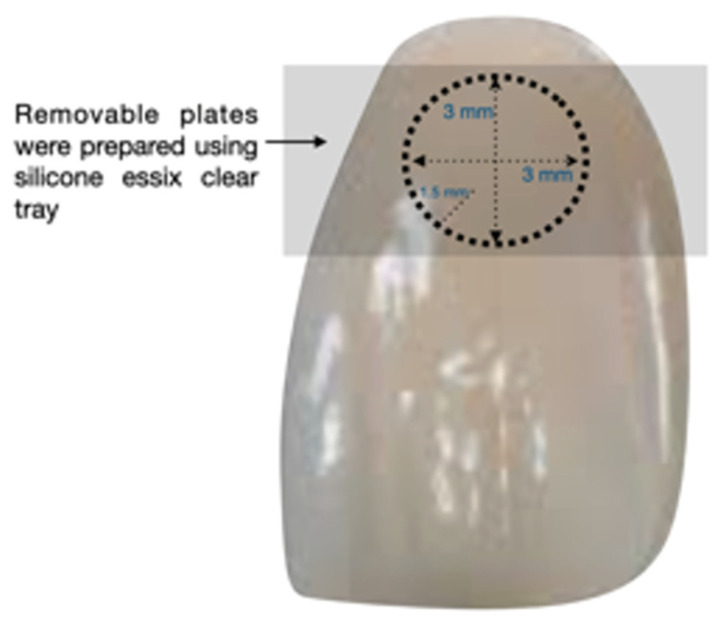
Representative image of standardized Class V cavity with the following dimensions: mesio-distal = 3.0 mm, cervical-occlusal = 3.0 mm, and depth = 1.5 mm with removable plates using a 1.0 mm width silicone Essix Clear tray (Dentsply, Charlotte, NC, USA) to standardize the color measurement surface area on the specimens. Filtek Bulk Fill was used to restore with either A1 or A3.

Forty Class V cavities on acrylic resin upper central incisor teeth were bur-prepared using a high-speed handpiece (Synea Vision TK 94, W&H Dentalwerk Bürmoos GmbH, Bürmoos, Salzburgo, Austria) with water coolant and a spherical diamond bur (bur head ø = 1.5 mm, # 1012, KG Sorensen, Barueri, SP, Brazil) to standardize the cavity preparation. The cavity preparations, restoration, and data analysis were executed by a single trained operator (F.F.). Removable plates were prepared using a silicone Essix clear tray (1.0 mm width of Essix C; Dentsply, FL, USA) on a vacuum press machine (Ministar, Scheu, Iserlohn, Nordrhein-Westfalen, Iserlohn, Germany) with standard windows (R = 3.0 mm) to standardize the preparations’ position on the specimens. Overall, the windows on the removable plates were used as a guide to standardize the bur preparations, dimensions were verified using a digital caliper (Teknikel, Istanbul, Turkey), and a periodontal probe was used to measure the following dimensions: mesio-distal = 3.0 mm, cervical–occlusal = 3.0 mm, and depth = 1.5 mm. The teeth were randomly assigned into groups of two different colors from the Vita Classical Shade Guide, A1 and A3, and restored with a combination of tested materials as indicated (Figure 1). Filtek Bulk Fill (3M Oral Care, St. Paul, MN, USA) was used to restore the samples, containing approx. 64.5 wt% or 42.5 vol% filler at sizes ranging from 0.01 to 5.0 µm based on manufacturer information.

After the preparations were completed, the cavities underwent cleaning using air/water spray and were dried with compressed air. Subsequently, a universal adhesive system (AdheSE Universal, Ivoclar Vivadent, Schaan, Liechtenstein, Germany) was applied to the cavities. The adhesive was spread and gently rubbed onto the surface for a duration of 20 s. An air stream was then directed onto the treated surface for 5 s to remove excess solvent following the guidelines provided by the manufacturer. Light curing was subsequently carried out for 20 s on the buccal surface using a Valo multiwave light-curing device (Ultradent, South Jordan, Utah, Salt Lake City, UT, USA) with a radiant power of 1000 mW/cm^2^ [23]. The radiant power was measured prior to use in each experimental group using a radiometer (Bluephase Meter II, Ivoclar Vivadent). All Class V restorations were restored with two oblique resin composite increments which were individually light-cured (20 J/cm^2^) for 20 s on the buccal surface. A mylar matrix was positioned over the second increment of resin composite to avoid any excess.

### 2.2. Shade Evaluation Methods

**Figure 2:**
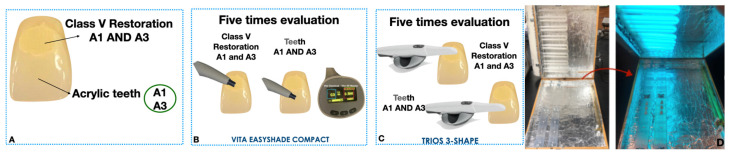
(**A**) Class V restoration in A1 and A3 at baseline. (**B**,**C**) Five repeated measurements were performed on top of the resin composite restoration and on top of the acrylic teeth in a randomized way and location under D65 illumination for VITA Easyshade and Trios T3. (**D**) Aging process chamber with UV-B exposure at 37 °C.

Shade analysis was conducted using digital devices, specifically the Vita Easyshade Compact device (Vita North America, Yorba Linda, CA, USA) and the Trios T3 Scanner intraoral scanner (3Shape, Copenhagen, Denmark), as depicted in Figure 2, immediately after restoration (i.e., baseline). All shade measurements were acquired within a standardized simulated daylight chamber (Model D65, Macbeth Judge II, Grands Rapids, MI, USA), employing a consistent white (L* = 49.07, a* = 6.51, and b* = 8.17) background (L* = 69.07, a* = 7.51, and b* = 9.17). Samples were consistently positioned in the same manner throughout the analysis. The background within the standardized simulated daylight chamber was uniformly maintained. Additionally, a consistent operator was maintained throughout the process to ensure standardization.

#### 2.2.1. Spectrophotometer (Vita Easyshade Compact, Vita North America, Yorba Linda, CA, USA) 

White balance calibration was performed to ensure the accuracy of measurements prior to measurements according to the manufacturer’s instructions. Subsequently, a small aperture (3.0 mm) was utilized, specifically tailored to the dimensions of Class V restorations. A systematic method for shade evaluation on Class V restorations was then executed using the spectrophotometer alongside detachable plates. Prior to each measurement, the spectrophotometer was calibrated to guarantee the consistent positioning of readings. The aperture comprehensively encompassed all regions of the Class V restoration. After completing the recording, the principal vestibular area was marked, and the 3D-MASTER shade was displayed. 

#### 2.2.2. Trios Intraoral (Trios T3 Scanner, 3Shape, Copenhagen, Denmark)

White balance calibration was performed to ensure the accuracy of measurements prior to measurements. The scanner’s operating system and the shade determination module were calibrated. The corresponding central maxillary incisor was scanned from the vestibular, incisal, and palatine aspects. After completing the recording, the principal vestibular area was marked, and the 3D-MASTER shade was displayed. Following all measurements, the 3D-MASTER values from the Easyshade device and the Trios T3s scanner were evaluated.

### 2.3. Shade Measurements

Five repeated measurements in each sample were conducted on top of the resin composite restorations in the shades A1 and A3, as well as on top of the acrylic teeth in the shades A1 and A3, in a randomized manner and location under D65 illumination using the VITA Easyshade device and Trios T3 (Figure 2A). Following all measurements, the 3D-MASTER shade guide values obtained from the Easyshade device and the Trios T3 scanner were used for shade comparison, and the percentage of shade trueness recorded was assessed (Figure 2B,C).

Following the initial shade measurement, artificial aging was conducted using an apparatus previously described [24,25]. This system comprises an enclosed box structure with eight tubular fluorescent lamps (ultraviolet light) situated in the upper section, maintained at a temperature of 37 °C. The lower section accommodates the specimens. The samples underwent UV light aging for three cycles: the first cycle spanned five days, equivalent to one year of clinical use for resin composites; the second cycle extended for an additional five days, corresponding to two years of clinical use; and the final cycle encompassed ten more days, representing five clinical years of UV-B exposure at 3 °C. After each cycle, new shade measurements were taken to assess the shade match under extended temperature conditions (Figure 2D). 

### 2.4. Data Analyses

The primary focus of data analysis was on the calculation of arithmetic means for the percentage of repeatability in color matching across two sets of dental shade samples, A1 and A3, based on the obtained 3D-MASTER shade. These analyses were conducted using two distinct assessment devices: the Trios scanner and the Easyshade spectrophotometer. The dataset comprised 20 samples, evenly divided into 10 samples of shade A1 and 10 samples of shade A3. The percentage of repeatability represents the closeness of the measured shade to the standard or target shade, indicating the accuracy of each device in shade matching. For each group of samples (A1 and A3, assessed by both the Trios and Easyshade devices), the arithmetic mean of repeatability percentages was determined. This was accomplished by summing the repeatability percentages recorded for the 10 samples within each category and dividing this total by the number of samples, 10. The calculation was performed separately for each device and shade category to ascertain the accuracy levels effectively. Consequently, the analysis yielded four distinct means as follows:

- The average percentage of trueness for shade A1 samples as assessed by the Trios scanner.
- The average percentage of trueness for shade A1 samples as assessed by the Easyshade spectrophotometer.
- The average percentage of trueness for shade A3 samples as assessed by the Trios scanner.
- The average percentage of trueness for shade A3 samples as assessed by the Easyshade spectrophotometer.

These mean values provide a quantifiable measure of each device’s effectiveness in achieving accurate shade matches, offering critical insights into their performance in replicating the designated dental shades, A1 and A3. These mean values were analyzed using a two-way ANOVA (SPSS version 25, IBM Corp., Armonk, NY, USA) with Tukey’s test for pairwise comparison (α = 0.05). A Kolmogorov–Smirnov test was used to confirm normality. 

## 3. Results

There was no statistically significant difference when comparing the shade measurement devices (*p* > 0.05). A slight difference was observed in the distributions of tooth shade recorded for the spectrophotometer (Vita Easyshade Compact, Vita North America, Yorba Linda, CA, USA) and intraoral (Trios T3 Scanner, 3Shape, Copenhagen, Denmark) scanner under D65 illumination. At baseline, five repeated measurements of each sample revealed that the percentage of trueness in matching the color assessed by the 3D-MASTER shade guide for both the intraoral scanner and spectrophotometer in artificial teeth shades A1 and A3 was 100%, with consistent 3D-MASTER shade guide color readings. However, when the shade for Class V resin composite restorations was measured, the intraoral scanner achieved 80% trueness, while the spectrophotometer showed 60% trueness in shades A1 and A3 after conducting five shade measurements of each sample (see Table 1).

After aging, five repeated measurements of each sample revealed that the percentage of trueness in matching the color assessed by the 3D-MASTER shade guide for both the intraoral scanner, the Trios T3s scanner, and the spectrophotometer, Vita Easyshade Compact, in artificial teeth in shade A1 readings was 80%. On the other hand, when the shade was recorded in Class V resin composite restorations, the intraoral scanner and spectrophotometer recorded 60% trueness after conducting five shade measurements of each sample. In artificial teeth in the shade A3, the intraoral scanner trueness was 60% in artificial teeth and Class V resin composite restorations, and the spectrophotometer showed 60% trueness in artificial teeth and 40% trueness in Class V resin composite restorations (Table 2).

## 4. Discussion

The findings of this in vitro study reveal that the intraoral Trios T3 scanner demonstrated reproducibility in shade determination compared to the Vita Easyshade Compact spectrophotometer. The experimental data invalidated the following corresponding research hypothesis: (1) shade matching in acrylic teeth and resin composite will not be significantly influenced by the method of analysis. Both the intraoral scanner and spectrophotometer are reliable for initial shade matching, but their accuracy decreases in long-term evaluation, particularly for darker shades. We focused here on Class V restorations given their increasing prevalence in many aging societies with the increased retention of dentition compared to that the past. A literature search revealed a scarcity of investigations into intraoral scanners available in the market for shade determination, even though their clinical application is significant. The Trios T3 scanner is a pioneer in developing shade determination software (Trios Color, 3Shape, Copenhagen, Denmark) using 3D-MASTER Shade Guide scales(VITA Zahnfabrik, Bad Sackingen, Germany), and several studies have compared this digital tool with visual methods and spectrophotometers [10,26,27,28,29,30]. However, the results of this present study might translate to other intraoral scanning technologies.

The following secondary hypothesis was accepted: (2) shade matching in acrylic teeth and resin composite will be significantly influenced by the method of shade analysis. The repeatability of the Trios T3 scanner for shade determination, when repeated five times, showed 100% trueness in artificial teeth in the shade A1 and A3. However, the results were better on artificial acrylic teeth than on Class V resin composite restorations, with slightly higher reproducibility (80%) corresponding to the Vita 3D-MASTER Shade Guide values compared to the spectrophotometer (60%) at baseline (Table 1). Therefore, different material surfaces could interfere with the scanner’s trueness shade determination. This result stands in contrast to a previous literature review, which analyzed articles published between 1 January 1985 and 1 January 2021. This review discussed various visual and digital shade selection methods, along with the factors and conditions affecting their accuracy and precision. According to this review, dental spectrophotometers were found to offer the highest overall accuracy and precision among different shade selection methods [29]. Generally, shade selection using scanners is influenced by several elements, including ambient light, image capture techniques, color-analyzing software, and the specific shade guide mode utilized [29].

After aging, the trueness percentage recorded by the Trios T3 scanner and spectrophotometer in artificial acrylic teeth in the shade A1 was 80%. For Class V resin composite restorations, both the scanner and spectrophotometer recorded 60% trueness. In artificial acrylic teeth in the shade A3, the scanner’s trueness was 60% in both artificial teeth and Class V restorations; the spectrophotometer showed 60% trueness in artificial teeth and 40% in Class V restorations. This implies that while the scanner is generally reliable, its precision might fluctuate for different shades. Previous clinical studies, including those by Brandt et al. [10] and Huang et al. [28], which evaluated tooth shade determination using the Trios T3 scanner and a spectrophotometer, are in agreement with the findings of the present study, revealing comparable effectiveness in methods of tooth shade analysis.

This study corroborates findings from a previous literature review that evaluated the accuracy, repeatability, and reproducibility of intraoral scanners in digital shade determination, revealing no significant difference between shade determination with the Trios T3 scanner versus visual shade determination in terms of accuracy and repeatability [31]. Despite variations in tooth shade selection using intraoral scanners, Abu-Hossin et al. demonstrated repeatability with the Trios T3 scanner and found moderate agreement when using the Cerec Omnicam device (CEREC, Dentsply Sirona, Charlotte, NC, USA) [27].

Another in vitro study focused on the repeatability and reproducibility of various intraoral scanners, including the Trios T3 scanner and the Cerec Omnicam device. This study found that the Cerec Omnicam device exhibited the lowest accuracy among the tested scanners [31]. In the context of shade matching, the literature has identified spectrophotometer measurements as the most reliable method for tooth shade determination. Spectrophotometers are praised for their fewer error sources, primarily due to their ease of handling, automated operation, and the ability to calibrate the instrument for each measurement, thereby minimizing potential errors [31]. However, with continuous advancements in scanner software, digital intraoral scanners are becoming increasingly reliable for shade determination in dentistry. The high precision of digital shade determination provided by these scanners could streamline the workflow in everyday dental practice and potentially replace conventional visual methods [30]. Emphasizing the critical role of shade selection, it is essential to consider the clinical implications of even minor inaccuracies in shade matching. Accurate shade matching is paramount in dentistry, as it directly impacts the aesthetic outcomes of dental restorations [32]. Minor deviations from the target shade can lead to restorations that are visually distinguishable from the surrounding natural teeth, potentially compromising patient satisfaction and the overall success of the dental procedure [33]. Furthermore, the challenge of achieving an exact match underscores the importance of utilizing advanced shade-matching technologies and devices, such as intraoral scanners and spectrophotometers [34,35,36,37]. These tools aim to minimize human error and enhance the accuracy of shade determination. However, as indicated by our research, the effectiveness of these devices can vary, highlighting the need for continuous improvement and the adaptation of these technologies to clinical needs.

In the present study, artificial aging was induced for 120 h, reflecting the equivalent of five clinical years, conducted using a patented aging machine (BR 10 2014 019793) comprising a box format [24]. The upper part of the box housed eight tubular-shaped fluorescent lamps (UV) with exposure to 37 °C, while the lower part reserved space for the samples [24,25]. The samples underwent aging under UV light for three cycles, with the last cycle lasting an additional ten days, corresponding to the equivalent of five clinical years [24,25]. A previous study has shown that this aging protocol had a discernible impact on the color matching of resin composites. An area for future research, which is not reflected when using the aging machine, is extrinsic staining and the effects it can have on shade. Additional research could also focus on the effect of the water-mediated degradation of resin composites and the effects on shade; experiments conducted herein were dry, which does not mimic the oral cavity.

In addition, a new material was used, with an enhanced depth of cure and minimal polymerization shrinkage, referred to as “bulk fill resin composites” [38]. In these new materials, increased light transmission is achieved by a modification in the filler content, an adjustment in filler size relative to the light wavelength, and an adaptation of the refractive index between the inorganic and organic fractions. Additionally, previous research recommends that when the high-viscosity versions of these materials are used, they should be preheated to improve their flowability, adaptability, wear resistance, and color stability [39]. As a result, the bulk fill resin composites can be placed in increments of up to 4.0 mm [40] to provide satisfactory mechanical properties while circumventing the disadvantages of the incremental technique [41].

The limitation of this in vitro study is that the intraoral scanner, being a clinical device, does not account for shade variation using the CIEDE2000 formula (∆E00), nor does it position the shades within the L, a, and b coordinates. However, the primary objective was to compare the repeatability of shade determination for resin composite restorations and acrylic teeth, simulating clinical scenarios of clinician color determination using digital tools to provide the 3D-MASTER shade guide scale. Another limitation of the present study was the absence of extremely high chroma shades such as A4 or B4. However, clinicians frequently opt to place resin composites in the A3 color when restoring cervical areas such as Class V cavities or resin composite veneers. Other factors like adherence to manufacturer instructions, complete photopolymerization, and operator skill may affect shade matching and the composite shade itself.

Despite technological advancements in shade selection, there is currently a lack of data regarding shade selection using intraoral scanners available on the market. Therefore, further research is crucial to continue enhancing shade determination methods, with the potential to replace traditional visual techniques. Additionally, in vivo studies are necessary to evaluate shade determination across variations in polished materials and to assess shade selection in different dental materials.

## 5. Conclusions

Within the limitations of this current study, the following was concluded: 

Intraoral scanners and spectrophotometers are reliable for baseline shade selection, but their accuracy decreased after aging, for which we simulated 5 clinical years of long-term treatment, particularly for darker shades. Clinicians should be aware of these limitations and consider them in the long-term color selection of resin composite restorations and acrylic teeth.

## Figures and Tables

**Table 1:** The shade difference between Delara Acrylic Teeth-T46 (Kulzer, South Blend, IN, USA) shade A1/A3 using spectrophotometer (Vita Easyshade Compact, Vita North America, Yorba Linda, CA, USA) and intraoral (Trios T3 Scanner, 3Shape, Copenhagen, Denmark) scanner at baseline.

	Trios		Vita Easyshade Compact	
Filtek Bulk Fill A1	Teeth A1	Class V	Teeth A1	Class V
	2L1.5	2M2	2L1.5	1M2
	2L1.5	3L1.5	2L1.5	2L1.5
	2L1.5	3L1.5	2L1.5	3L1.5
	2L1.5	3L1.5	2L1.5	3L1.5
	2L1.5	3L1.5	2L1.5	3L1.5
Filtek Bulk Fill A3	Teeth A3	Class V	Teeth A3	Class V
	3L1.5	3L1.5	3L1.5	2L1.5
	3L1.5	2M2	3L1.5	1M2
	3L1.5	2M2	3L1.5	2M2
	3L1.5	2M2	3L1.5	1M2
	3L1.5	2M2	3L1.5	1M2

**Table 2:** The shade difference between Delara Acrylic Teeth—T46 (Kulzer LLC, South Blend, IN, USA) shade A1/A3 using spectrophotometer (Vita Easyshade Compact, Vita North America, Yorba Linda, CA, USA) and intraoral (Trios T3 Scanner, 3Shape, Copenhagen, Denmark) scanner after artificial aging.

	Trios		Vita Easyshade Compact	
Filtek Bulk Fill A1	Teeth A1	Class V	Teeth A1	Class V
	2L1.5	4L1.5	2L1.5	4L1.5
	2L1.5	4M1	2L1.5	3L1.5
	3L1.5	3L1.5	3L1.5	4L1.5
	2L1.5	3L1.5	2L1.5	4L1.5
	2L1.5	3L1.5	2L1.5	3L1.5
Filtek Bulk Fill A3	Teeth A3	Class V	Teeth A3	Class V
	3L1.5	4L1.5	3L1.5	4L1.5
	3L1.5	3M2	3L1.5	3M2
	4L1.5	3M2	4L1.5	3M2
	3L1.5	4L1.5	3L1.5	3L1.5
	4L1.5	4L1.5	4L1.5	3L1.5

## Data Availability

The data presented in this study are available on request from the corresponding author.
